# The association of baseline N-terminal pro-B-type natriuretic peptide with short and long-term prognosis following percutaneous coronary intervention in non-ST segment elevation acute coronary syndrome with multivessel coronary artery disease: a retrospective cohort study

**DOI:** 10.1186/s12872-021-02010-9

**Published:** 2021-04-21

**Authors:** Wen-fei He, Lei Jiang, Yi-yue Chen, Yuan-hui Liu, Peng-yuan Chen, Chong-yang Duan, Li-huan Zeng, Hua-lin Fan, Xue-biao Wei, Wei Guo, Wei Chen, Jun Li, Wen-sheng Li, Zhi-qiang Guo, Zhi-kai Liu, Ning Tan, Ji-yan Chen, Peng-cheng He

**Affiliations:** 1 Department of Cardiology, Guangdong Cardiovascular Institute, Guangdong Provincial Key Laboratory of Coronary Heart Disease Prevention, Guangdong Provincial People’s Hospital, Guangdong Academy of Medical Sciences, Guangzhou, 510100 China; 2 Department of Cardiology, Guangdong Provincial People’s Hospital’s Nanhai Hospital, The Second People’s Hospital of Nanhai District, Foshan, 528000 China; 3 Department of Cardiology, Fuwai Hospital Chinese Academy of Medical Sciences, Shenzhen, 518000 China; 4 Department of Biostatistics, School of Public Health, Southern Medical University, Guangzhou, 510100 China; 5 School of Medicine, The Second School of Clinical Medicine, Southern Medical University, Guangzhou, 510100 China; 6 School of Medicine, Guangdong Provincial People’s Hospital, South China University of Technology, Guangzhou, 510100 China; 7 Department of Cardiology, Fujian Provincial Clinical College of Fujian Medical University, Fujian Provincial Hospital, Fujian Institute of Cardiovascular Disease, Fuzhou, 350000 China; 8 Department of Cardiovascular Medicine, Zhuhai People’s Hospital, Zhuhai, 519000 China; 9 Department of Cardiology, Shunde Hospital, Southern Medical University, Foshan, 528000 China

**Keywords:** N-terminal pro-B-type natriuretic peptide, Non-ST segment elevation acute coronary syndrome, Multivessel coronary artery disease

## Abstract

**Background:**

Several studies have shown that N-terminal pro-B-type natriuretic peptide (NT-proBNP) is strongly correlated with the complexity of coronary artery disease and the prognosis of patients with non-ST segment elevation acute coronary syndrome (NSTE-ACS), However, it remains unclear about the prognostic value of NT-proBNP in patients with NSTE-ACS and multivessel coronary artery disease (MCAD) undergoing percutaneous coronary intervention (PCI). Therefore, this study aimed to reveal the relationship between NT-proBNP levels and the prognosis for NSTE-ACS patients with MCAD undergoing successful PCI.

**Methods:**

This study enrolled 1022 consecutive NSTE-ACS patients with MCAD from January 2010 to December 2014. The information of NT-proBNP levels was available from these patients. The primary outcome was in-hospital all-cause death. In addition, the 3-year follow-up all-cause death was also ascertained.

**Results:**

A total of 12 (1.2%) deaths were reported during hospitalization. The 4th quartile group of NT-proBNP (> 1287 pg/ml) showed the highest in-hospital all-cause death rate (4.3%) (*P* < 0.001). Besides, logistic analyses revealed that the increasing NT-proBNP level was robustly associated with an increased risk of in-hospital all-cause death (adjusted odds ratio (OR): 2.86, 95% confidence interval (CI) = 1.16–7.03, *P* = 0.022). NT-proBNP was able to predict the in-hospital all-cause death (area under the curve (AUC) = 0.888, 95% CI = 0.834–0.941, *P* < 0.001; cutoff: 1568 pg/ml). Moreover, as revealed by cumulative event analyses, a higher NT-proBNP level was significantly related to a higher long-term all-cause death rate compared with a lower NT-proBNP level (*P* < 0.0001).

**Conclusions:**

The increasing NT-proBNP level is significantly associated with the increased risks of in-hospital and long-term all-cause deaths among NSTE-ACS patients with MCAD undergoing PCI. Typically, NT-proBN* P* > 1568 pg/ml is related to the all-cause and in-hospital deaths.

## Background

Individuals with multivessel coronary artery disease (MCAD) account for approximately 40–70% of all non-ST segment elevation acute coronary syndrome (NSTE-ACS) patients who undergo coronary angiography [1–3]. Currently, percutaneous coronary intervention (PCI) has been considered as an option for the treatment of MCAD and left main (LM) disease, which is ascribed to its higher procedural success rates and comparable benefits [4–7]. However, the incidence rates of cardiovascular morbidity and mortality after PCI remain high in this population [8, 9]. Thus, it is important to develop a prognostic biomarker to identify the high-risk patients.

Several studies have shown that the plasma levels of natriuretic peptides, such as B-type natriuretic peptide (BNP) and N-terminal pro-B-type natriuretic peptide (NT-proBNP), are robustly related to the prognosis for NSTE-ACS patients [10–12]. Furthermore, previous studies have demonstrated that the increased NT-proBNP levels among NSTE-ACS patients are independently associated with the presence of more complex and severe coronary lesions [13–15]. However, it remains unclear about the prognostic value of NT-proBNP in patients with NSTE-ACS and MCAD undergoing PCI. This study aimed to investigate the relationship between NT-proBNP levels and the short-term prognosis for NSTE-ACS patients with MCAD.

## Methods

### Study design

More details about the cohort were presented in our previous research [16], which was designed to detect the association between parenteral anticoagulation therapy and the clinical outcomes of NSTE-ACS patients undergoing PCI. In brief, altogether 8197 NSTE-ACS patients undergoing PCI were enrolled from 5 centers from January 1st, 2010 to December 31st, 2014. Specifically, the patient inclusion criteria were as follows, patients aged 18 years or older who were diagnosed with MCAD, those whose NT-proBNP levels were determined on the first day of admission, and those with cardiac arrest and return of circulation. MCAD was defined as lesions with ≥ 50% diameter stenosis in the LM artery or ≥ 2 major coronary vessels with ≥ 50% stenosis. The patient exclusion criteria were shown below: the pregnant patients or those with missing baseline NT-proBNP levels were excluded from this study. The enrolled patients were divided into 4 groups based on the quartiles of NT-proBNP level. Ultrasonic cardiography was performed after admission, and the left ventricular ejection fraction (LVEF) was calculated by Simpson's biplane method. The estimated glomerular filtration rate (eGFR) was calculated by the Modification of Diet in Renal Disease equation among the Chinese patients [17]. Our study protocol was approved by the Central Ethics Committee of Guangdong Provincial People’s Hospital (Guangzhou, China). This study was conducted in accordance with the Declaration of Helsinki.

### Data collection

Data were obtained from the first interview when the patients were admitted to the hospital. Baseline characteristic data, including demographic data and medical history, were recorded by the responsible nurse or doctor. The procedural information was obtained from the catheterization report. The laboratory examinations for all patients were carried out during the first 24 h after admission before the procedure was performed, and the NT-proBNP levels were measured by the electrochemiluminescence immunoassay (Roche Diagnostics, Germany). All patients received the drug eluting stent. All interventional strategies were selected at the discretion of the heart team. In-hospital and follow-up assessments were performed by means of clinic visits or telephone interviews from November 7th, 2015 to December 30st, 2016.

### Outcomes

The primary outcome was in-hospital all-cause death. The secondary outcomes were all-cause death during the 3-year follow-up and in-hospital major adverse cardiovascular events (MACE), which were defined as a composite of all-cause death, myocardial infarction (MI) and stroke. The definitions of all clinical complications assessed during the follow-up period were the same as the original registry [16]. Death was defined as all-cause death, regardless of the cardiac or non-cardiac origin, according to death records. According to the third version of Universal Definition of Myocardial Infarction, MI was defined as classical symptoms accompanied by the elevation of cardiac injury biomarker. Stroke was defined as the presence of a new focal neurologic deficit of vascular origin, with signs or symptoms lasting for over 24 h. The clinical events committee evaluated all clinical outcomes independently.

### Statistical analysis

Statistical analysis was implemented using SAS version 9.4 (SAS Institute, Cary, NC, USA). Continuous variables were presented as mean ± standard deviation (SD) and compared by Student’s t-test (parametric variables). Categorical variables were expressed as absolute and relative frequencies. The predictive value of NT-proBNP for different clinical outcomes was evaluated by multivariate regression analyses, and it was included as a continuous variable after logarithmic transformation. All the confounders included in the final model were the clinical important factors or were significant in univariate analyses. The log NT-proBNP, Anaemia, Chronic heart failure, Chronic kidney disease, NSTEMI, LVEF and age were incorporated into the final model for death analysis; whereas log NT-proBNP, Anaemia, Chronic heart failure, chronic kidney disease, NSTEMI, LVEF, age, Diabetes, Myocardial infarction and operation time were incorporated into the final model for MACE analysis. Moreover, receiver-operating characteristic (ROC) curves were plotted to assess the ability of NT-proBNP in discriminating surviving patients from those who died during hospitalization. Also, the Youden index was utilized to determine the best cutoff of NT-proBNP level in predicting all-cause death, and this level was expected to be used in further analyses. Further, cumulative event analyses were performed to compare the long-term prognosis between patients divided by the best cutoff level of NT-proBNP. All P-values < 0.05 were considered statistically significant.

## Results

### Baseline characteristics

Among the 1022 patients who met the final inclusion and exclusion criteria, 118 (11.5%) were female, with the average age of 65.8 (standard difference: 10.5; range, 33–90) years. All the enrolled patients were of Han nationality. There were 585 patients older than 65 years in total. Variables were compared by baseline NT-proBNP quartile values. The baseline characteristics are presented in Table 1. It was observed that, patients with high NT-proBNP levels were older, with lower body weight (BW), higher heart rates, as well as more frequent non-ST segment elevation myocardial infarction (NSTEMI), chronic kidney disease (CKD), anaemia diabetes and stroke than those with low NT-proBNP levels. In addition, chronic heart failure, prior MI, prior PCI and lower LVEF were more frequent in the higher NT-proBNP quartiles. Furthermore, most of the treatment variables did not show significant differences among the different NT-proBNP groups, except for the operation time.

**Table 1:** Baseline characteristics of different baseline NT-proBNP levels

Baseline characteristics	NT-proBNP (Q1 < 96 pg/ml) N = 257	NT-proBNP (96 pg/ml < Q2 < 328 pg/ml) N = 255	NT-proBNP (328 pg/ml < Q3 < 1287 pg/ml) N = 255	NT-proBNP (Q4 > 1287 pg/ml) N = 255	P value
**General characteristics**					
Mean age (SD), y	61.02 ± 10.11	65.05 ± 10.25	66.94 ± 10.45	70.09 ± 8.90	< 0.001
Age ≥ 65y, No. (%)	103 (40.1)	134 (52.5)	158 (62.0)	190 (74.5)	< 0.001
Female, No. (%)	61 (23.7)	53 (20.8)	72 (28.2)	76 (29.8)	0.076
Weight, mean (SD), kg	68.29 ± 11.59	67.47 ± 12.29	65.91 ± 13.49	62.28 ± 13.89	< 0.001
Heart rate, mean (SD), bpm	74.38 ± 10.27	72.79 ± 10.77	75.15 ± 12.69	80.66 ± 16.02	< 0.001
LVEF, mean (SD), %	67.60 ± 5.09	65.21 ± 7.55	59.59 ± 11.10	49.85 ± 14.66	< 0.001
Anaemia, No. (%)	49 (19.1)	65 (25.5)	98 (38.4)	148 (58.0)	< 0.001
Serum creatinine level, mean (SD), μmol/dL	0.93 ± 0.25	0.99 ± 0.26	1.10 ± 0.48	1.60 ± 1.60	< 0.001
**Disease type, No. (%)**					
NSTEMI	44 (17.1)	64 (25.3)	83 (32.5)	126 (49.4)	< 0.001
Unstable angina	213 (82.9)	189 (74.7)	172 (67.5)	129 (50.6)	NA
**eGFR, mL/min/1.73m**^**2**^					
Mean (SD),	88.77 ± 23.41	82.22 ± 23.63	76.04 ± 27.49	59.53 ± 25.68	< 0.001
≤ 60 mL/min/1.73m^2^, No. (%)	27 (10.5)	42 (16.5)	68 (26.7)	133 (52.2)	< 0.001
**Risk factors and prior medical history, No. (%)**					
Current smoker	74 (28.8)	71 (27.8)	80 (31.4)	69 (27.1)	0.726
Hypertension	169 (65.8)	183 (71.8)	171 (67.1)	183 (71.8)	0.316
Diabetes	89 (34.6)	80 (31.4)	92 (36.1)	120 (47.1)	0.002
Cardiac arrest	0 (0.0)	0 (0.0)	1 (0.4)	1 (0.4)	0.570
Chronic heart failure	18 (7.0)	21 (8.2)	46 (18.0)	107 (42.0)	< 0.001
Myocardial infarction	23 (8.9)	28 (11.0)	67 (26.3)	74 (29.0)	< 0.001
Percutaneous coronary intervention	64 (24.9)	42 (16.5)	35 (13.7)	49 (19.2)	0.009
Coronary artery bypass surgery	2 (0.8)	3 (1.2)	2 (0.8)	4 (1.6)	0.796
Stroke	8 (3.1)	23 (9.0)	32 (12.5)	35 (13.7)	< 0.001
**Treated lesion, No. (%)**					
LM	25 (9.7)	31 (12.2)	35 (13.8)	36 (14.2)	0.402
LAD	161 (62.6)	170 (66.9)	168 (66.4)	163 (64.4)	0.730
LCX	92 (35.8)	117 (46.1)	121 (47.8)	99 (39.1)	0.017
RCA	120 (46.7)	120 (47.2)	102 (40.3)	101 (39.9)	0.181
Multivessel intervention	122 (47.5)	139 (54.7)	132 (52.2)	109 (43.1)	0.044
Completeness of revascularization	59 (23.0)	69 (27.2)	55 (21.7)	33 (13.0)	0.001
**Drug eluting stent type, No. (%)**					
First generation	122 (53.0)	131 (60.4)	132 (62.6)	144 (74.6)	< 0.001
Second generation	108 (47.0)	86 (39.6)	79 (37.4)	49 (25.4)	NA

*NT-proBNP* N-terminal pro-B-type natriuretic peptide, *Q* quartile, *NSTEMI* non–ST-segment elevation myocardial infarction, *LVEF* left ventricular ejection fraction, *eGFR* estimated glomerular filtration rate, *NA* not applicable, *LM* left main, *LAD* left anterior descending, *LCX* left circumflex, *RCA* right coronary artery

### In-hospital outcomes

Altogether 12 (1.2%) in-hospital all-cause deaths were reported. Typically, patients with NT-proBNP > 1287 pg/ml had the highest all-cause death rate (4.3% highest vs. 0.0% lowest, *P* < 0.001, Table 2). Patients in the highest quartile of NT-proBNP had the highest in-hospital MACE rate (6.3% highest vs 0.8% lowest, *P* < 0.001), but there was no significant difference in in-hospital stroke or MI between different groups (Table 2). The higher NT-

**Table 2:** Clinical outcomes of different baseline NT-proBNP levels

In-hospital outcomes	NT-proBNP (Q1 < 96 pg/ml) N = 257	NT-proBNP (96 pg/ml < Q2 < 328 pg/ml) N = 255	NT-proBNP (328 pg/ml < Q3 < 1287 pg/ml) N = 255	NT-proBNP (Q4 > 1287 pg/ml) N = 255	P-value
All-cause death	0 (0.0%)	0 (0.0%)	1 (0.4%)	11 (4.3%)	< 0.001
Stroke	0 (0.0%)	0 (0.0%)	2 (0.8%)	3 (1.2%)	0.142
MI	2 (0.8%)	0 (0.0%)	2 (0.8%)	3 (1.2%)	0.434
MACE	2 (0.8%)	0 (0.0%)	4 (1.6%)	16 (6.3%)	< 0.001

*MACE* major adverse cardiovascular events, *MI* myocardial infarction, *NT-proBNP* N-terminal pro-B-type natriuretic peptide

**Fig. 1:**
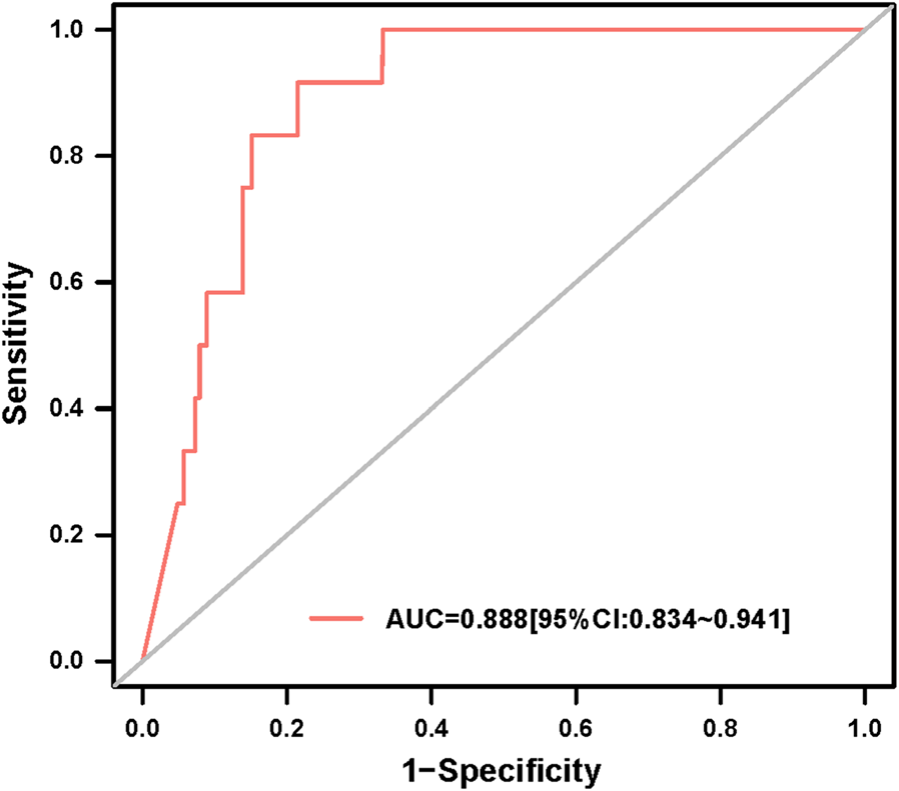
ROC analysis of NT-proBNP in predicting in-hospital all-cause death; AUC: area under the curve; CI: confidence interval

proBNP level was associated with a higher risk of in-hospital all-cause death (univariate: odd ratio (OR): 3.06, 95% confidence interval (CI): 1.77–5.28, *P* < 0.001; multivariate: adjusted OR: 2.86, 95% CI: 1.16–7.03, *P* = 0.022) (Table 3). Additionally, the increasing NT-proBNP level was related to an increased risk of in-hospital MACE (adjusted OR: 2.09, 95% CI: 1.35–3.23, *P* = 0.001) after adjusting for confounders. Additionally, according to discrimination analyses, NT-proBNP was adequate for predicting the in-hospital all-cause death (Fig. 1). The area under the ROC curve (AUC) was 0.888 (95% CI: 0.834–0.941, *P* < 0.001). The best cutoff level of NT-proBNP in predicting in-hospital death was 1568 pg/ml (sensitivity: 91.7%, specificity: 78.5%).

**Table 3:** Univariate and multivariate analyses of in-hospital outcomes

	Univariate analysis			Multivariate analysis		
	OR	95% C.I	P	OR	95% C.I	P
**All-cause death**						
log NT-proBNP	3.06	1.77–5.28	< 0.001	2.86	1.16–7.03	0.022
Anaemia	9.43	2.05–43.27	0.004	4.63	0.49–43.41	0.179
Chronic heart failure	3.14	0.99–10.01	0.053	0.88	0.20–3.93	0.865
Chronic kidney disease	3.98	1.25–12.64	0.019	1.96	0.33–11.5	0.458
NSTEMI	3.15	0.99–10.01	0.051	3.07	0.56–16.90	0.198
LVEF	0.96	0.92–1.00	0.037	1.00	0.95–1.05	0.990
Age	1.04	0.98–1.11	0.170	2.86	1.16–7.03	0.436
**MACE**						
log NT-proBNP	1.98	1.47–2.68	< 0.001	2.09	1.35–3.23	0.001
Anaemia	2.72	1.15–6.42	0.023	1.09	0.37–3.18	0.875
Chronic heart failure	1.28	0.47–3.51	0.633	0.39	0.11–1.36	0.139
Chronic kidney disease	2.86	1.23–6.68	0.015	1.73	0.58–5.19	0.326
NSTEMI	1.27	0.53–3.07	0.589	0.74	0.26–2.11	0.571
LVEF	0.98	0.95–1.01	0.130	1.01	0.97–1.05	0.617
Age	1.04	0.99–1.08	0.096	1.00	0.95–1.05	0.940
Diabetes	1.41	0.60–3.30	0.425	0.92	0.34–2.51	0.868
Myocardial infarction	1.64	0.63–4.25	0.308	0.81	0.25–2.70	0.736
Operation time 24–72 h (Reference is 24 h)	0.93	0.33–2.62	0.884	0.82	0.23–2.90	0.758
Operation time > 72 h (Reference is 24 h)	1.46	0.54–3.98	0.456	1.62	0.52–5.03	0.406

*NT-proBNP* N-terminal pro-B-type natriuretic peptide, *NSTEMI* non–ST-segment elevation myocardial infarction, *LVEF* left ventricular ejection fraction, *MACE* major adverse cardiovascular events

### Long-term outcomes

**Fig. 2:**
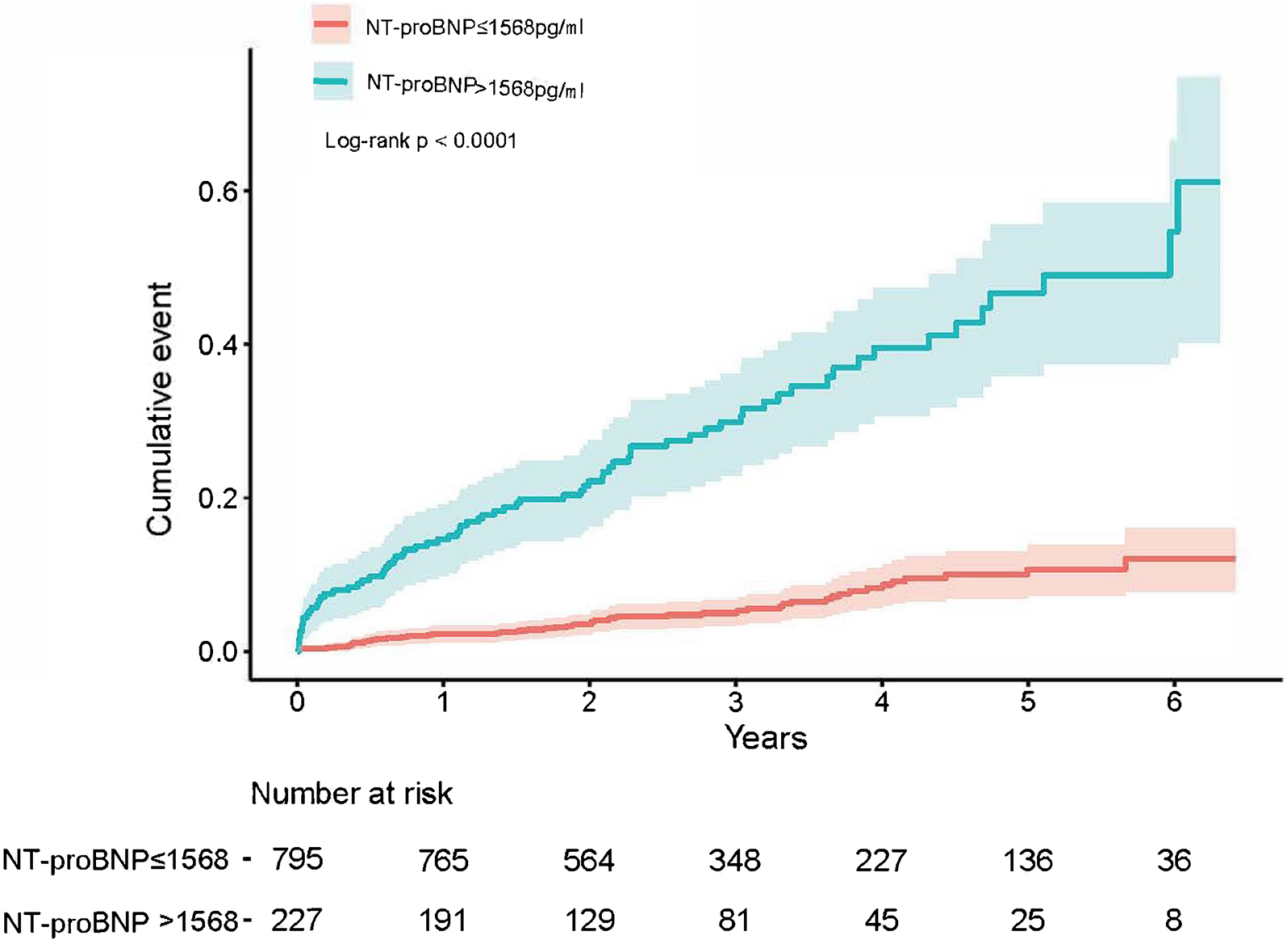
Cumulative event analysis during follow-up between patients with high and low levels of NT-pro BNP (cutoff: 1568 pg/ml)

All the 1022 patients were followed up for 3 years. Deaths were recorded in 121 (11.8%) patients. Thereafter, the long-term all-cause death was compared between patients with baseline NT-proBNP ≤ 1568 pg/ml and those with baseline NT-proBNP > 1568 pg/ml. As a result, patients with higher NT-proBNP levels showed a significantly higher long-term event rate than those with lower NT-proBNP levels (*P* < 0.0001) (Fig. 2).

## Discussion

This study discovered that patients with NT-proBNP levels > 1287 pg/ml showed the highest in-hospital all-cause death and MACE rates. In addition, this study determined the optimal cutoff of 1568 pg/ml to evaluate in-hospital death. During the 3-year follow-up period, patients with higher NT-proBNP levels (> 1568 pg/ml) had a higher all-cause death rate.

Findings in this study showed that the increased NT-proBNP level was related to the adverse outcomes of NSTE-ACS patients with MCAD. However, the pathophysiological mechanism underlying the association between ischaemia and NT-proBNP elevation remains unknown yet. It has been reported in previous studies that, myocardial ischaemia can cause transient and permanent increase in wall stress, myocardial tension, and subsequently induce BNP neurohormone release from the ventricular myocardium [18–20]. Furthermore, several studies [20–22] have revealed the up-regulation of ventricular BNP genes upon myocardial hypoxia, thus provoking an increase in the plasma NT-proBNP level. In addition, the NT-proBNP level is strongly related to cardiac function, which can be used to detect left ventricular (LV) systolic and diastolic dysfunction [23, 24]. An immediate increase in plasma BNP level may occur after myocardial ischaemia [9, 25] and before the elevation of traditional markers for myocardial necrosis. Meanwhile, the increase magnitude of BNP level is proportional to the severity of myocardial ischaemia [26]. In this study, patients with NT-proBNP levels greater than 1287 pg/ml presented with the highest LV dysfunction level (mean LVEF, 49.85 ± 14.66). Therefore, it was speculated that the higher NT-proBNP level on admission was the combined result of both myocardial ischaemia before the index event and the index event itself. Another mechanism underlying the NT-proBNP elevation among patients with acute coronary syndromes (ACS) was the permanently elevated NT-proBNP level that reflected ventricular dysfunction or heart failure before the index event. Interestingly, patients in NT-proBNP > 1287 pg/ml group had near-normal LVEF (49.85% ± 14.66%), so it was surmised that these patients experienced a certain form of diastolic dysfunction, since previous study found that NT-proBNP plasma levels increased in patients with diastolic dysfunction [27].

Laurenz Jaberg et al*.* demonstrated that the plasma NT-proBNP level was a strong predictor for outcome in patients undergoing acute LM coronary artery stenting. However, this was a retrospective study, and the NT-proBNP level was measured only in 71 ACS patients with LM disease upon hospital admission [11]. Our study extended this interaction to patients with NSTE-ACS and MCAD, another population at high risk of ischemia. MCAD predisposes to a more severe and extensive myocardial ischaemia, which results in the higher NT-proBNP level.

Cardiovascular diseases are associated with several endocrine situations, like diabetes, hypothyroidism and so on [28, 29]. In this study, the highest quartile of NT-proBNP which had the highest mortality and MACE also had the highest proportion of diabetes. Generally, diabetes is regarded as a risk factor of cardiovascular evens, and several mechanisms, including ubiquitin proteasome system, adiponectin, carbonic anhydrase, has been established to clarify the relationship between diabetes and the initiation and progression of atherosclerosis, restenosis after PCI and myocardial remolding [28, 30–32].

NT-proBNP is positively associated with age [33]. In this research, the group with a higher level of NT-proBNP also had an older age. Age, together with gender, are two important factors that influence the disease distribution and outcomes, [34, 35]which may affect our results.

To remove the influence of confounders like diabetes and age, multivariate regression analyses were carried out and the result is the same both before and after the adjustment. As for gender, there is no significant difference between these four groups.

Nevertheless, NT-proBNP was still robustly associated with death after adjusting for LVEF in this study. Previous studies have demonstrated that the association between NT-proBNP and death is not linked to LVEF [36, 37]. Furthermore, the NT-proBNP level is previously found to be associated not only with myocardial ischaemia in coronary heart disease (CHD), but also with all kinds of cardiac pathological conditions, such as activation of the renin–angiotensin–aldosterone system [38]. Besides, tissue hypoxia is found in previous studies to induce the release of BNP in the absence of LV dysfunction [39]. In this population, patients with higher NT-proBNP levels upon admission have a greater extent of myocardial ischaemia because of the more severe coronary lesions and subclinical LV dysfunction, possibly consequent to the effects of chronic repetitive ischaemia on the myocardium [40, 41]. All these mechanisms may result in poor prognosis and an increased risk of death. However, more investigations are warranted to further study the exact mechanism underlying the connection between NT-proBNP and death. However, the casual relationship between the NT-proBNP and CV death cannot be confirmed due to the study design, and further basic researches were warranted to demonstrated the potential mechanism.

## Limitations

Some limitations should be noted in this study. Firstly, although great efforts were made, we were still unable to adjust for all the potential confounders due to the retrospective study design. The impact of treatment changes over time was not determined as well. Secondly, the exact cause of death was not determined due to an unavailability of first-hand clinical documents, making it difficult for us to determine the causal relationship between death and the increasing NT-proBNP. Thirdly, due to the lack of persistent monitoring of in-hospital NT-proBNP, this study was unable to determine a relationship between changes in NT-proBNP level and prognosis. Therefore, more detailed studies are warranted.

## Conclusion

Findings in this study suggest that, a high level of NT-proBNP on admission is associated with a higher risk of in-hospital all-cause death among NSTE-ACS patients with MCAD undergoing PCI. Moreover, NT-proBNP > 1568 pg/ml is a reasonable cutoff value related to the in-hospital and long-term all-cause deaths.

## Data Availability

The datasets used and/or analysed during the current study available from the corresponding author on reasonable request.

## Abbreviations

BNP: B-type natriuretic peptide
eGFR: Estimated glomerular filtration
LM: Left main
LV: Left ventricular
LVEF: Left ventricular ejection fraction
MACE: Major adverse cardiovascular events
MCAD: Multivessel coronary artery disease
NT-proBNP: N-terminal pro-B-type natriuretic peptide
NSTE-ACS: Non-ST segment elevation acute coronary syndrome
PCI: Percutaneous coronary intervention
ROC: Receiver-operating characteristic
